# Fecal microbiome-based clustering identifies distinct disease subtypes with differing metabolomic profiles and treatment outcomes in canine chronic enteropathy

**DOI:** 10.1080/01652176.2026.2744638

**Published:** 2026-10-29

**Authors:** Junjie Li, Takahiro Aoki, Shiho Miyata, Atsushi Fukaya, Yuhei Hayashi, Hidehiro Hirao, Shin Yoshimoto, Kentaro Nagaoka

**Affiliations:** a Laboratory of Veterinary Physiology, Cooperative Department of Veterinary Medicine, Tokyo University of Agriculture and Technology, Tokyo, Japan; b Biotics Research Institute, Morinaga Milk Industry Co., Ltd., Kanagawa, Japan; c Japan Animal Referral Medical Center Kawasaki, Kanagawa, Japan

**Keywords:** Dog, chronic enteropathy, gut microbiota, dysbiosis, fecal metabolomics, treatment response, kynurenic acid, partitioning around medoids

## Abstract

Canine chronic enteropathy (CE) is a heterogeneous disorder with variable clinical outcomes. We aimed to identify fecal microbiome and metabolomic signatures associated with CE severity and treatment outcomes. Fecal samples from dogs with CE (*n* = 56), acute diarrhea (*n* = 8), and healthy controls (*n* = 126) were analyzed by 16S rRNA gene sequencing and GC-MS-based untargeted metabolomics. CE dogs exhibited significantly reduced microbial diversity compared with the other groups, and unsupervised Partitioning Around Medoids clustering identified two microbiome clusters within the CE cohort. Cluster 2 was characterized by dysbiosis, with enrichment of Streptococcus, Enterococcus, and Klebsiella, and depletion of short-chain fatty acid producers, including Fusobacterium and Blautia. Among dogs with baseline Canine Chronic Enteropathy Clinical Activity Index (CCECAI) > 3, 10 of 12 Cluster 1 dogs (83.3%) achieved remission (CCECAI ≤ 3) after treatment, compared with 7 of 18 Cluster 2 dogs (38.9%; *p* = 0.0256). CCECAI scores decreased significantly after treatment in Cluster 1 (*p* = 0.0005) but not in Cluster 2 (*p* = 0.077). Fecal propanoic acid and kynurenic acid levels also differed between clusters. These findings suggest that gut microbiota and associated metabolites may contribute to differences in CE severity and treatment outcomes.

## Introduction

Canine chronic enteropathy (CE) is a gastrointestinal disorder characterized by persistent or recurrent digestive symptoms lasting more than three weeks, with primary clinical signs including diarrhea, vomiting, and anorexia (Allenspach and Mochel 2022). CE is conventionally classified by response to stepwise therapeutic interventions into food-responsive enteropathy (FRE), microbiota-responsive enteropathy (MRE), immunosuppressant-responsive enteropathy (IRE), and non-responsive enteropathy (NRE) (Dandrieux and Mansfield 2019; Rodrigues et al. 2025). However, these therapeutic-response categories do not consistently correspond to disease severity, histopathological findings, or underlying etiology (Dandrieux 2016; Jergens and Heilmann 2022), and classification can only be determined retrospectively after sequential treatment trials. This limitation highlights the need for complementary stratification approaches that can characterize disease phenotypes at the time of diagnosis.

The gut microbiota constitutes a dynamic gastrointestinal ecosystem that plays a critical role in host immune development, metabolic homeostasis, and numerous physiological processes (Pilla and Suchodolski, 2020; Gacesa et al. 2022; Shoubridge et al. 2022). Its effects are mediated through immune stimulation, modulation of intestinal architecture, competitive exclusion of pathogens, and production of key metabolites such as short-chain fatty acids (SCFAs) (Guard et al. 2015). Understanding the complex interactions among the host, gut microbiota, and their metabolic end products provides important insights into the pathophysiology of gastrointestinal disorders. Analogous to human inflammatory bowel disease (IBD) (Franzosa et al. 2019; Andoh and Nishida 2022), canine enteropathies are characterized by marked dysbiosis, including reduced microbial diversity, structural alterations in community composition, and shifts in core taxa, accompanied by changes in fecal metabolomic profiles (Dandrieux 2016; Fritsch et al. 2022; Kaga et al. 2024; Chen et al. 2025).

Building on these observations, non-invasive diagnostic approaches leveraging gut microbiome signatures have been proposed as promising tools for IBD detection in humans (Zheng et al. 2024), and several studies have suggested microbiome-based diagnostic criteria applicable to canine CE (AlShawaqfeh et al. 2017; Jergens and Heilmann 2022). However, CE is a multifactorial condition influenced by age, breed, diet, clinical history, and individual variability in disease presentation (Galler et al. 2022; Jergens and Heilmann 2022; Yu et al. 2023). Because non-invasive methods often provide limited prognostic information, identifying microbiome and metabolomic features that reflect disease severity and predict treatment outcomes remains a priority for improving clinical management.

The primary objective of this study was therefore to characterize gut microbiota profiles associated with canine CE, to stratify CE cases into biologically meaningful subgroups based on microbiome composition, and to explore the metabolomic correlates of these subgroups and their potential association with disease severity and treatment outcomes. Dogs with acute diarrhea, presenting to a primary care clinic with acute-onset gastrointestinal signs, were included as a comparator group to distinguish CE-specific chronic dysbiosis from transient microbiome perturbations associated with acute gastrointestinal disease.

## Materials and methods

### Study population and sampling

Between January 2022 and December 2023, fecal samples from dogs with chronic enteropathy (CE group), acute diarrhea (AD group), and clinically healthy controls (HC group) were analyzed in this study. The samples were originally collected as part of independent research projects conducted by Tokyo University of Agriculture and Technology (TUAT) and Morinaga Milk Industry Co., Ltd., respectively.

56 client-owned dogs presenting with clinical signs of chronic gastrointestinal disease were recruited from the Japan Animal Referral Medical Center (JARMEC). Dogs were diagnosed with chronic enteropathy (CE group) by attending veterinarians based on comprehensive assessment of clinical signs, complete blood count, serum biochemistry, abdominal ultrasonography, endoscopic biopsy, and evaluation of housing conditions, dietary and medication history, and previous illnesses. Extra-gastrointestinal causes of chronic gastrointestinal signs, including hypoadrenocorticism, exocrine pancreatic insufficiency, chronic pancreatitis, and primary infectious enteropathies, were excluded based on these clinical findings; specific hormonal testing was performed when clinically indicated. Disease severity was graded using the Canine Chronic Enteropathy Clinical Activity Index (CCECAI; Allenspach et al. 2007). The CE study protocol was reviewed and approved by the Animal Experiment and Research Ethics Committee of the Faculty of Agriculture, Tokyo University of Agriculture and Technology (approval no. 0020008), as well as by the Ethics Committee of Morinaga Milk Industry Co., Ltd. (approval no. 21-001).

Fecal samples from dogs with acute diarrhea (AD group; *n* = 8) and clinically healthy dogs (HC group; *n* = 126) were originally collected as part of a research project conducted by Morinaga Milk Industry Co., Ltd. Fecal samples from the AD group were obtained from client-owned dogs diagnosed with acute diarrhea by veterinarians at Oda Animal Hospital. Acute diarrhea was defined as the presence of gastrointestinal signs lasting less than three weeks. Detailed classification of diarrhea type (hemorrhagic vs non-hemorrhagic) was not available from the clinical records. All dogs in the AD group were privately owned, lived in diverse household environments, and were fed various commercial diets. Fecal samples from the HC group were obtained from dogs housed at multiple commercial breeding facilities during routine management procedures. The AD and HC study protocol was reviewed and approved by the Ethics Committee of Morinaga Milk Industry Co., Ltd. (approval no. 21-004).

For the present study, fecal samples obtained from the TUAT-led CE study and the Morinaga-led AD/HC study were integrated and analyzed together as a secondary analysis to investigate fecal microbiome and metabolomic profiles across the three clinical groups. No additional animal interventions or sample collections were performed specifically for this study. All fecal samples were stored at −80 °C until analysis. Written informed consent was obtained from all dog owners or facility managers prior to sample collection.

### Fecal DNA extraction and 16S rRNA sequencing

Fecal DNA was extracted as previously described (Komatsu et al. 2022). Briefly, 500 μL of fecal sample in GuSCN solution was lysed with glass beads (300 mg, 0.1 mm diameter) using a FastPrep-24 5 G homogenizer (MP Biomedicals LLC, Santa Ana, CA, USA) at speed setting 5 for 45 s, with 5 min cooling intervals on ice. After centrifugation at 12,000 rpm for 5 min, DNA was extracted from 200 μL of supernatant using the GENE PREP STAR PI-480 instrument (Kurabo Industries Ltd., Osaka, Japan) according to the manufacturer's protocol. The V3–V4 region of the bacterial 16S rRNA gene was paired-end sequenced on the Illumina NextSeq 1000 platform using the NextSeq 1000/2000 P1 Reagent Kit (600 cycles; Illumina, San Diego, CA, USA).

Sequence data were analyzed using QIIME2 version 2022.8 (Bolyen et al. 2019). Demultiplexed reads were processed through filtering, denoising, merging, and chimera removal, and amplicon sequence variants (ASVs) were generated using DADA2 (Callahan et al. 2016). Taxonomic assignment was performed against the Greengenes2 database version 2022.10 (McDonald et al. 2024). Alpha diversity indices and Bray–Curtis dissimilarity-based principal coordinate analysis (PCoA) were calculated within QIIME2.

### Gut microbiota clustering

Clustering of gut microbiota profiles was performed at the genus level using R version 4.3.2 (R Foundation for Statistical Computing, Vienna, Austria) with the packages philentropy version 0.9.0 (Drost 2018), cluster version 2.1.8.1 (Maechler et al. 2019), and fpc version 2.2-13 (Hennig 2024). Jensen–Shannon distance (JSD) matrices were computed between samples, and Partitioning Around Medoids (PAM) clustering was applied. The optimal number of clusters was determined using the Calinski–Harabasz index.

### Fecal sample preparation for untargeted metabolomic analysis

Thirty milligrams of fecal sample were weighed into a 2 mL microtube, and 15 mg of 0.2 mm glass beads and 150 μL of PBS were added. The mixture was homogenized twice at 5,500 rpm for 20 s and centrifuged at 21,000 × g for 5 min at 4 °C. The supernatant was filtered through a 0.2 μm cellulose acetate membrane filter (ϕ13 mm; Toyo Roshi Kaisha, Ltd., Tokyo, Japan). Fifty microliters of filtered supernatant were combined with 250 μL of a ternary solvent system of methanol, chloroform, and water (2.5:1:1; Fujifilm Wako, Osaka, Japan) and 5 μL of 1 mg/mL 2-isopropylmalic acid (Fujifilm Wako) as an internal standard. The mixture was incubated at 1,200 rpm and 37 °C for 30 min, then centrifuged at 16,000 × g at 4 °C for 5 min. From 225 μL of the resulting supernatant, 200 μL of distilled water was added, and after a further centrifugation step at 16,000 × g for 5 min at 4 °C, 250 μL of supernatant was collected. Solvent was partially removed using a centrifugal evaporator CVE-2200 (Tokyo Rikakikai Co., Ltd., Tokyo, Japan) for 25 min. Concentrates were frozen at −80 °C and subsequently lyophilized for 16 h using a vacuum freeze dryer FDU-1200 (Tokyo Rikakikai Co., Ltd.). Lyophilized samples were reconstituted in 40 μL of 20 mg/mL methoxamine hydrochloride (Fujifilm Wako) in pyridine (Fujifilm Wako) and incubated at 1,200 rpm and 30 °C for 90 min. Subsequently, 20 μL of N-methyl-N-trimethylsilyl-trifluoroacetamide (MSTFA; Thermo Fisher Scientific, Waltham, MA, USA) was added, and samples were incubated at 1,200 rpm and 37 °C for 45 min to complete derivatization.

### Untargeted GC-MS analysis

GC-MS analysis was performed as previously described with minor modifications (Li et al. 2024). One microliter of each derivatized sample was injected into a GC-MS QP2020 NX system (Shimadzu Corporation, Kyoto, Japan) equipped with an AOC-20i autosampler (Shimadzu Corporation). Chromatographic separation was performed on a DB-5 capillary column (1.00 μm film thickness; Agilent Technologies, Inc., Santa Clara, CA, USA). The injector temperature was maintained at 280 °C with a constant carrier gas flow of 5 mL/min. Electron ionization was performed at 70 eV, and mass spectra were acquired every 0.2 s over an m/z range of 45–600. The oven temperature program was as follows: initial hold at 100 °C for 4 min, ramp at 10 °C/min to 320 °C, and final hold at 320 °C for 11 min. Metabolite identification was performed using MS-DIAL version 4.90 in combination with the Shimadzu Smart Metabolite Database (OA_TMS_DB5_37min_V3_Scan; Shimadzu Corporation). Retention indices derived from an *n*-alkane series supported peak alignment and annotation. Data processing parameters were set to an m/z range of 45–600 Da, retention index tolerance of 20, and mass-to-charge tolerance of 0.5 Da, with minimum EI spectral similarity and overall identification score thresholds of 70. Raw peak intensities were normalized to the internal standard signal, and when multiple silylated derivatives were detected for a single metabolite, only the most abundant derivative was retained for analysis.

### Fecal SCFA quantification by GC-MS

Fecal short-chain fatty acids (SCFAs; formate, acetate, propionate, butyrate, and pentanoate) were quantified by GC-MS as previously described with minor modifications (Furuhashi et al. 2018). Briefly, 35 mg of fecal sample was weighed into a 2 mL microtube with 15 mg of 0.2 mm glass beads and 1 mL of 10% isobutanol. The mixture was homogenized twice at 5,500 rpm for 20 s and centrifuged at 21,000 × g for 5 min at 4 °C. From 675 μL of supernatant, 20 μL of 3-methylpentanoate (internal standard), 125 μL of 20 mM NaOH, and 400 μL of chloroform were added. After vortexing and centrifugation at 21,000 × g for 2 min at 4 °C, 400 μL of the upper phase was transferred to a fresh tube, and 70 μL isobutanol, 100 μL pyridine, and 80 μL DNase/RNase-free distilled water were added. Fifty microliters of isobutyl chloroformate was added with the cap open for 3 min to allow gas to escape, followed by addition of 150 μL hexane. After vortexing and centrifugation at 21,000 × g for 2 min at 4 °C, 80 μL of the upper organic phase was transferred to a GC vial for analysis.

GC-MS measurements were performed by injecting 1 μL of derivatized sample into a GC-MS QP2020 NX system equipped with an HP-5MS capillary column (30 m × 0.25 mm i.d., 0.25 μm film thickness; Agilent Technologies, Inc.). The injector temperature was set at 250 °C with a carrier gas flow of 5 mL/min. The oven temperature program consisted of an initial hold at 50 °C for 5 min, a ramp at 5 °C/min to 150 °C, a ramp at 40 °C/min to 330 °C, and a final hold at 330 °C for 1 min. Mass spectra were acquired over an m/z range of 30–600 in electron ionization mode at 70 eV. SCFA concentrations were determined from extracted ion chromatogram peak areas normalized to the internal standard.

### Statistical analysis

Intergroup comparisons of alpha diversity indices and bacterial relative abundances were performed using the Kruskal–Wallis test followed by the Mann–Whitney U test, with Bonferroni correction applied for multiple comparisons. Beta diversity was assessed by permutational multivariate analysis of variance (PERMANOVA) using the R package psych version 2.5.6 (Makowski 2018) and SPSS version 30.0 (IBM Corp., Armonk, NY, USA). Linear discriminant analysis effect size (LEfSe) was performed using the R package microeco version 1.15.0 (Liu et al. 2025). For metabolomic analyses, principal component analysis (PCA) was performed in R version 4.5.1. Differential metabolites between groups were identified using thresholds of |log₂ fold change| > 1 and false discovery rate (FDR) < 0.05, calculated by the Benjamini–Hochberg procedure. Clinical and demographic characteristics (age, body weight, serum albumin, CRP, and pre-medication status) were compared between clusters using the Mann–Whitney U test for continuous variables and Fisher's exact test for categorical variables. Because CE dogs received diverse and individualized treatments between initial and follow-up assessments, treatment response was evaluated solely on the basis of CCECAI change, irrespective of the specific therapeutic regimen employed. Treatment outcome analyses were restricted to dogs with both pre- and post-treatment CCECAI data available and a baseline CCECAI > 3, to exclude individuals already in remission at initial presentation and to enable meaningful assessment of treatment response. Remission was defined as CCECAI ≤ 3 at follow-up; an additional analysis using ≥ 75% CCECAI reduction as an alternative response criterion was also performed. Differences in remission rates between clusters were assessed using Fisher's exact test. Pre- and post-treatment CCECAI scores within each cluster were compared using the Wilcoxon signed-rank test. Post-treatment CCECAI scores between clusters were compared using the Mann–Whitney U test. The association between cluster assignment and baseline CCECAI severity was evaluated using cumulative logit ordinal logistic regression adjusted for age, sex, and neuter status. To assess the robustness of the HC group comparisons, a sensitivity analysis was performed excluding puppies aged ≤ 3 months (*n* = 29) from the HC group. A *p*-value < 0.05 was considered statistically significant throughout.

## Results

### Clinical characteristics

A total of 56 dogs diagnosed with CE were enrolled, along with 8 dogs diagnosed with acute diarrhea (AD group) and 126 clinically healthy dogs (HC group). Detailed individual characteristics of all groups are provided in Supplementary Tables S1–S3. The most common breeds in the CE group were Shiba Inu (*n* = 8), mixed breed (*n* = 7), toy poodle (*n* = 4), and dachshund (*n* = 3). Among the 56 CE dogs, 33 were female (29 spayed), 22 were male (14 neutered), and 1 was not recorded; the mean age at initial presentation was 8.20 ± 3.52 years (*n* = 55, excluding one dog with unrecorded age). Median baseline serum albumin was 2.10 g/dL (IQR: 1.80–2.80), and 18 of 54 dogs with available data (33.3%) had hypoalbuminemia (albumin < 2.0 g/dL). Median baseline CRP was 0.42 mg/dL (IQR: 0.14–1.39). Twenty-three of 56 dogs (41.1%) had received steroids/immunosuppressants or antibiotics from their primary care veterinarians before referral and baseline fecal sampling.

### Gut microbiota and fecal metabolomic profiles differed among healthy, acute diarrhea, and chronic enteropathy groups

A total of 3,180,943 sequence reads were obtained from 193 samples (mean ± SD: 16,481 ± 11,572 reads per sample), yielding 1,166 ASV features. Significant differences in both Chao1 and Shannon alpha diversity indices were observed among the three groups, with the HC group showing significantly higher values than both the AD and CE groups (Figure 1A). Principal coordinate analysis (PCoA) based on Bray–Curtis dissimilarity at the species-level annotation provided by the Greengenes2 revealed clear separation between the HC group and both the AD and CE groups (Figure 1B; PERMANOVA F = 22.658, R^2^ = 0.193, *p* = 0.001). At the phylum level, the dominant taxa across all groups were *Firmicutes_A*, *Firmicutes_D*, *Bacteroidota*, *Fusobacteriota*, *Firmicutes_C*, *Actinobacteriota*, and *Proteobacteria* (Figure 1C). At the genus level, *Streptococcus* was most abundant in the CE group, whereas *Fusobacterium_A*, *Fusobacterium_B*, *Faecalibacterium*, *Peptacetobacter*, and *Ligilactobacillus* were more prevalent in the HC group (Figure 1D).

**Figure 1.**
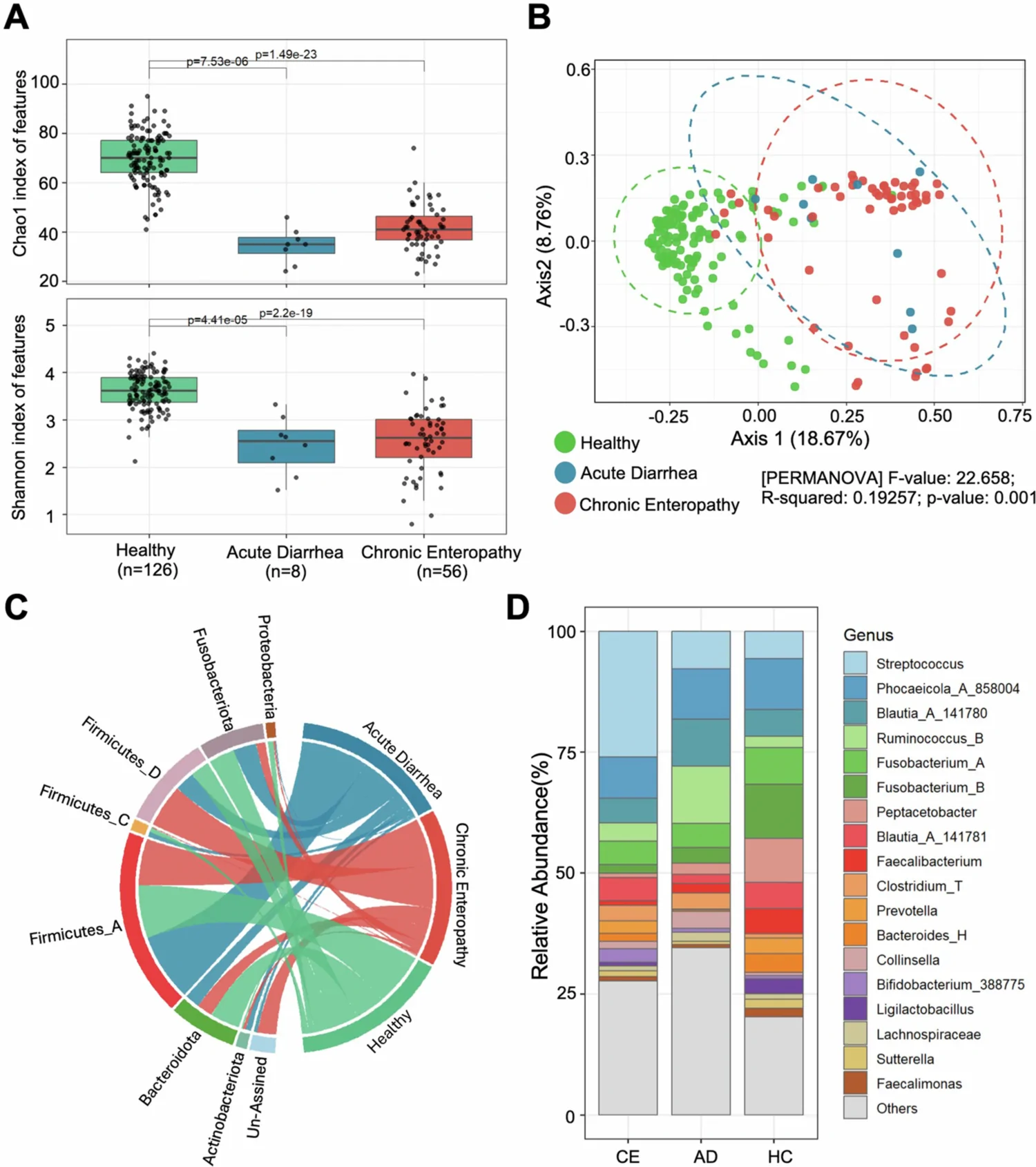
(A) Comparison of fecal microbiota *α*-diversity (Chao1 index, upper; Shannon index, lower) among healthy dogs (HC), dogs with acute diarrhea (AD), and dogs with chronic enteropathy (CE). Group differences were assessed using the Kruskal–Wallis test with Bonferroni correction. (B) Principal coordinate analysis (PCoA) of fecal microbiota *β*-diversity based on Bray–Curtis dissimilarity at the species level. Pairwise comparisons were assessed by PERMANOVA with Bonferroni correction. (C) Relative abundance of major bacterial phyla across groups. (D) Relative abundance of the top 20 most abundant genera across groups.

For metabolomic analysis, 44 fecal samples from the CE group and 7 from the AD group were available. A total of 206 metabolites were identified and classified according to Human Metabolome Database superclasses (Figure 2A). PCA did not reveal clear separation between the AD and CE groups (Figure 2B), likely reflecting the heterogeneity within the CE cohort. Nevertheless, volcano plot analysis identified 42 metabolites that differed significantly between the two groups (Figure 2C), prompting further investigation of metabolomic variation within the CE group through microbiome-based subgrouping.

**Figure 2.**
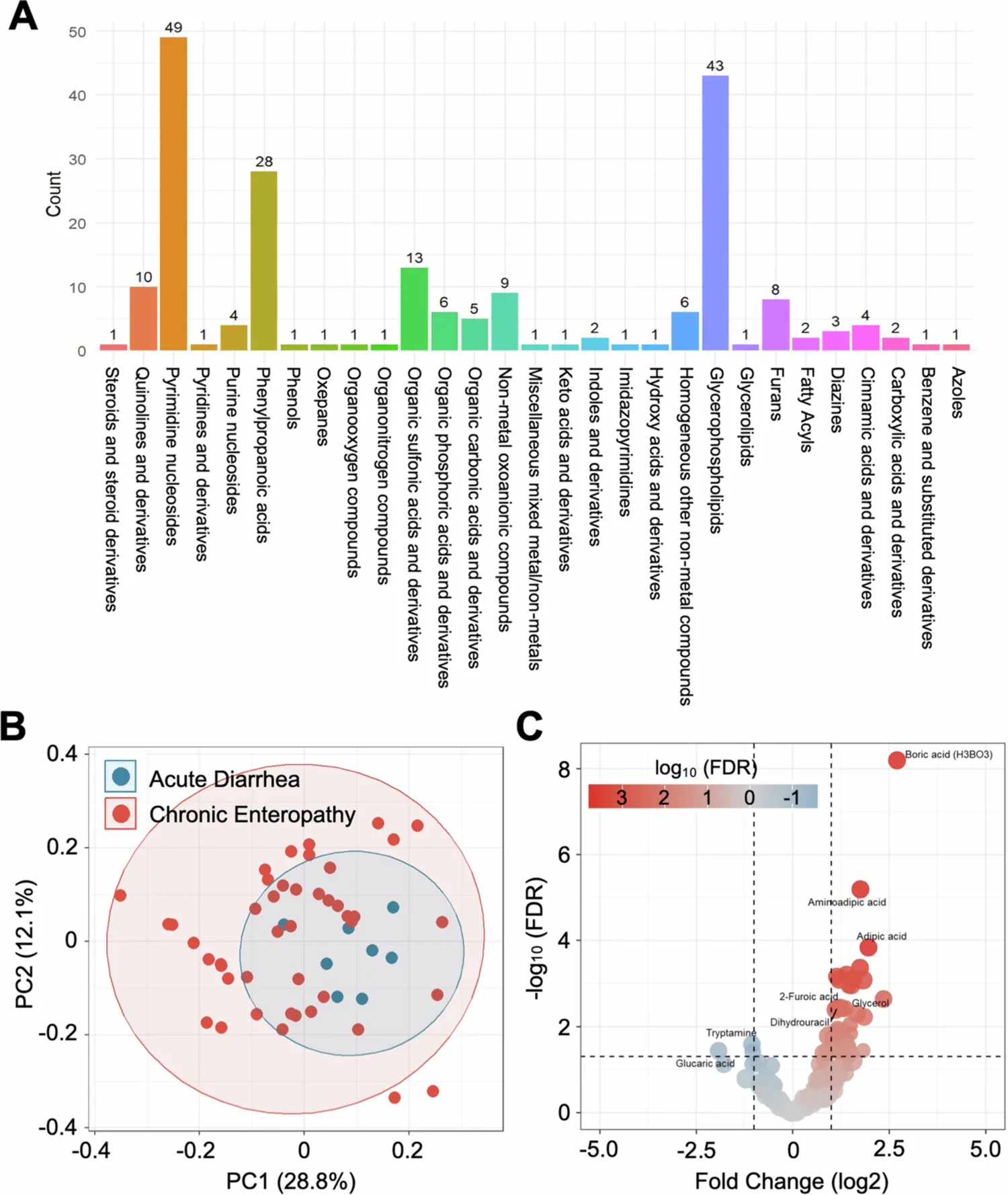
(A) Classification of identified fecal metabolites based on Human Metabolome Database superclasses. (B) PCA plot illustrating variance in the fecal metabolome between the AD and CE groups. (C) Volcano plot showing metabolites that differed significantly between the AD and CE groups. Dashed lines indicate thresholds of |log₂ fold change| > 1 and FDR < 0.05.

### PAM clustering identifies two distinct microbiome subtypes within the chronic enteropathy group

To investigate heterogeneity in gut microbiome composition among CE dogs, PAM clustering was applied at the genus level using Jensen–Shannon distances. Based on the highest Calinski–Harabasz index (Supplementary Figure S1), all CE individuals were assigned to either Cluster 1 (*n* = 22) or Cluster 2 (*n* = 34) (Figure 3A). Ordinal logistic regression adjusting for age, sex, and neuter status showed that Cluster 2 dogs tended to have higher baseline CCECAI scores compared with Cluster 1, although this did not reach statistical significance (OR = 1.79, 95% CI: 0.60–5.38, *p* = 0.298; Figure 3B). Clinical and demographic characteristics, including age, sex, body weight, serum albumin, CRP, and pre-medication status, did not differ significantly between clusters (Table 1). Neither age, sex, nor neuter status was significantly associated with baseline CCECAI severity, suggesting that the observed difference in disease severity between clusters was not confounded by these demographic factors. To assess treatment response while excluding individuals already in remission at initial presentation, analyses were restricted to dogs with baseline CCECAI > 3 and available post-treatment data (Cluster 1: *n* = 12; Cluster 2: *n* = 18; Supplementary Table S3). Of these, 10 of 12 Cluster 1 dogs (83.3%) achieved remission (CCECAI ≤ 3) following treatment, compared with 7 of 18 Cluster 2 dogs (38.9%; Fisher's exact test, OR = 7.86, 95% CI: 1.31–47.05, *p* = 0.0256; Table 2 and Figure 3C). CCECAI scores were significantly reduced following treatment in Cluster 1 (median change: −4.5, IQR: −7.0 to −4.0; Wilcoxon signed-rank test, *p* = 0.0005) but not in Cluster 2 (median change: −3.0, IQR: −5.8 to 0.8; *p* = 0.077), and post-treatment CCECAI scores were significantly lower in Cluster 1 than in Cluster 2 (median: 1.0 vs 6.0; Mann–Whitney U test, *p* = 0.0152; Figure 3C). When ≥ 75% CCECAI reduction was used as an alternative response criterion, a consistent trend was observed (Cluster 1: 7/12, 58.3% vs Cluster 2: 6/18, 33.3%), although this did not reach statistical significance (Fisher's exact test, *p* = 0.264). The median interval between baseline and follow-up CCECAI assessments was 14 days (IQR: 12–15; range: 7–60). Body weight, serum albumin, CRP, and pre-medication status at baseline did not differ significantly between clusters in the treatment analysis subset (Supplementary Table S4), indicating that the observed differences in treatment outcomes were not attributable to baseline clinical or medication imbalances. The distribution of treatment categories (steroids/immunosuppressants, antibiotics, probiotics, and other medications) administered between baseline and follow-up assessments did not differ significantly between clusters in the treatment analysis subset (all *p* > 0.05; Table 1 and Supplementary Table S4), indicating that the observed differences in treatment outcomes were not attributable to differential treatment regimens.

**Table 1.** Clinical and demographic characteristics of CE dogs stratified by microbiome cluster.

		Cluster 1 (*n* = 22)	Cluster 2 (*n* = 34)	*p*-value
Age (years)	Median (IQR)	6.0 (4.0–10.0)	9.0 (7.0–11.0)	0.114
Sex	Female/Male	10/11	23/11	0.166
Body weight (kg)	Median (IQR)	5.50 (3.70–9.70)	6.45 (4.00–8.60)	0.763
Serum albumin (g/dL)	Median (IQR)	2.30 (1.90–3.10)	2.10 (1.80–2.70)	0.150
Hypoalbuminemia	Albumin < 2.0 g/dL	6/21 (28.6%)	12/33 (36.4%)	0.768
CRP (mg/dL)	Median (IQR)	0.52 (0.15–2.16)	0.41 (0.21–1.22)	0.690
Baseline CCECAI	Median (IQR)	4.0 (2.8–7.2)	6.0 (4.0–12.0)	0.131
Pre-medication^a^	Yes	10/22 (45.5%)	13/34 (38.2%)	0.781
Mediation^b^	S/I	16/21 (76.2%)	27/33 (81.8%)	0.733
	AB	13/21 (61.9%)	22/33 (66.7%)	0.775
	PB	1/21 (4.8%)	4/33 (12.1%)	0.638
	other	10/21 (47.6%)	15/33 (45.5%)	1.000

Continuous variables compared using Mann–Whitney U test; categorical variables compared using Fisher's exact test.

^a^ Pre-medication: dogs receiving steroids/immunosuppressants (S/I) or antibiotics (AB) before baseline fecal sampling.

^b^ Medication administered between initial and follow-up CCECAI assessments; dogs may fall into multiple categories. NR cases excluded from denominators.

Data shown as median (IQR) or n/total (%). BW, body weight; ALB, serum albumin; CRP, C-reactive protein; CCECAI, Canine Chronic Enteropathy Clinical Activity Index.

**Table 2.** Treatment outcomes in dogs with chronic enteropathy and baseline CCECAI > 3, stratified by microbiome cluster.

		Cluster 1 (*n* = 12)	Cluster 2 (*n* = 18)
CCECAI at initial presentation	Mild (4–5)	5	6
	Moderate (6–8)	4	4
	Severe (9–11)	1	1
	Very severe (≥12)	2	7
Median CCECAI (IQR)		7.0 (4.0–8.2)	7.5 (5.0–13.0)
CCECAI at follow-up	Insignificant (0–3)	10	7
	Mild (4–5)	1	2
	Moderate (6–8)	1	2
	Severe (9–11)	0	2
	Very severe (≥12)	0	5
Median CCECAI (IQR)		1.0 (0.0–3.0)	6.0 (1.2–12.5)
CCECAI change^a^ median (IQR)		−4.5 (−7.0 to −4.0)	−3.0 (−5.8 to 0.8)
Remission rate^b^	CCECAI ≤ 3 at follow-up	10/12 (83.3%)*	7/18 (38.9%)*
Treatment response^c^	Pre vs post (Wilcoxon)	*p* = 0.0005^†^	*p* = 0.0773 (ns)
Post-treatment CCECAI^d^	Group comparison (Mann–Whitney)		*p* = 0.0152^‡^

Only dogs with both pre- and post-treatment CCECAI data available and baseline CCECAI > 3 were included in this analysis (Cluster 1: *n* = 12; Cluster 2: *n* = 18). CCECAI, Canine Chronic Enteropathy Clinical Activity Index. IQR, interquartile range. ns, not significant.

^^a^^ CCECAI change = post − pre. Negative values indicate improvement.

^^b^^ Remission defined as CCECAI ≤ 3 at follow-up.

^^c^^ Wilcoxon signed-rank test comparing pre- and post-treatment CCECAI within each cluster.

^^d^^ Mann–Whitney U test comparing post-treatment CCECAI between clusters.

^*^ *p* = 0.0256 (Fisher’s exact test).

^^†^^ *p* < 0.05.

^^‡^^ *p* < 0.05.

**Figure 3.**
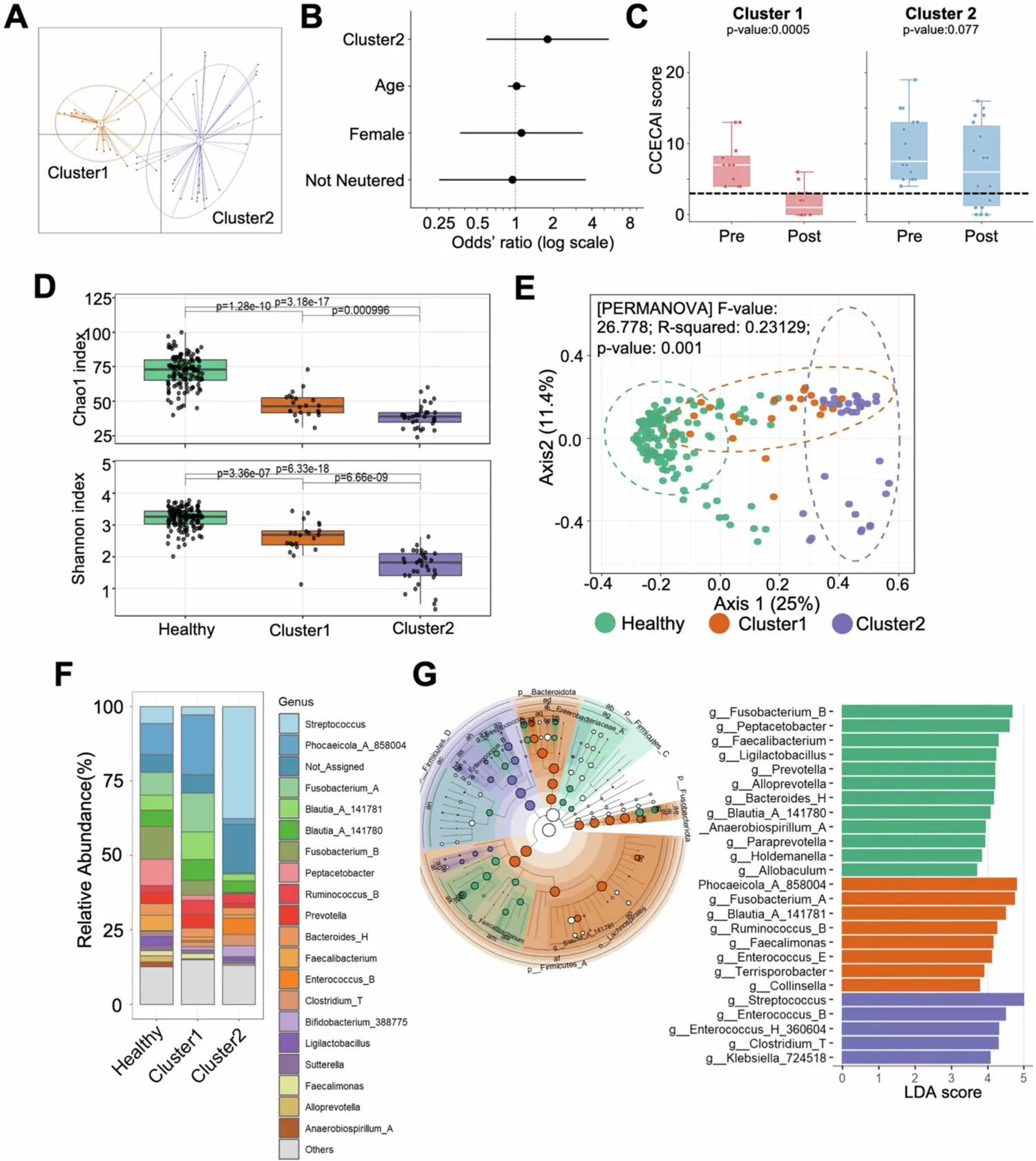
(A) Biplot of PAM clustering results based on fecal microbiota profiles at initial presentation, showing Cluster 1 (*n* = 22) and Cluster 2 (*n* = 34). (B) Association between initial CCECAI severity grade and cluster assignment, assessed by cumulative logit ordinal logistic regression adjusted for age, sex, and neuter status (OR = 1.79, 95% CI: 0.60–5.38, *p* = 0.298). Continuous variable (age) is expressed per one-year increment. Reference categories: Cluster 1, male, and neutered. (C) Paired dot plot showing individual pre- and post-treatment CCECAI scores in Cluster 1 (*n* = 10) and Cluster 2 (*n* = 18), restricted to dogs with baseline CCECAI > 3. Lines connect pre- and post-treatment values for each dog; blue, improved; orange, worsened; grey, no change. The dashed line indicates CCECAI = 3 (remission threshold). *p*-values are from the Wilcoxon signed-rank test. (D) Comparison of fecal microbiota *α*-diversity (Chao1 index, upper; Shannon index, lower) among the HC group, Cluster 1, and Cluster 2, assessed by the Kruskal–Wallis test with Bonferroni correction. (E) PCoA of fecal microbiota *β*-diversity among the HC group, Cluster 1, and Cluster 2. Pairwise comparisons were assessed by PERMANOVA with Bonferroni correction. (F) Relative abundance of the top 20 most abundant genera across the HC group, Cluster 1, and Cluster 2. (G) Cladogram and LDA score bar plot from LEfSe analysis showing key discriminative taxa between Cluster 1 and Cluster 2; the logarithmic LDA score threshold was set at 3.5.

Alpha diversity was significantly lower in both Cluster 1 and Cluster 2 compared with the HC group, with Cluster 2 showing the lowest values for both Chao1 and Shannon indices (Figure 3D). Beta diversity analysis confirmed significant differences in microbial community structure among the HC group, Cluster 1, and Cluster 2 (Figure 3E; PERMANOVA F = 26.778, R^2^ = 0.231, *p* = 0.001). At the genus level, Cluster 2 was characterized by a markedly higher relative abundance of *Streptococcus* compared with both the HC group and Cluster 1, and lower relative abundances of *Phocaeicola_A_858004*, *Fusobacterium_A*, *Blautia_A_141781*, *Blautia_A_141780*, *Fusobacterium_B*, and *Peptacetobacter* (Figure 3F). LEfSe analysis identified *Streptococcus*, *Enterococcus_H_360604*, *Clostridium_T*, and *Klebsiella* as key contributors to the dysbiotic profile of Cluster 2, while *Fusobacterium_A*, *Blautia_A_141781*, *Ruminococcus_B*, *Faecalimonas*, and *Enterococcus_E* were characteristic of Cluster 1 (Figure 3G).

### Fecal metabolomic profiles differed between Cluster 1 and Cluster 2

PCA demonstrated clear separation between Cluster 1 and Cluster 2 in metabolomic space (Figure 4A). Volcano plot analysis identified multiple metabolites that differed significantly between the two clusters (Figure 4B). Among the targeted SCFAs, propanoic acid concentration was significantly lower in Cluster 2 compared with Cluster 1, whereas formic, acetic, and butyric acid concentrations did not differ significantly between clusters (Figure 4C).

**Figure 4.**
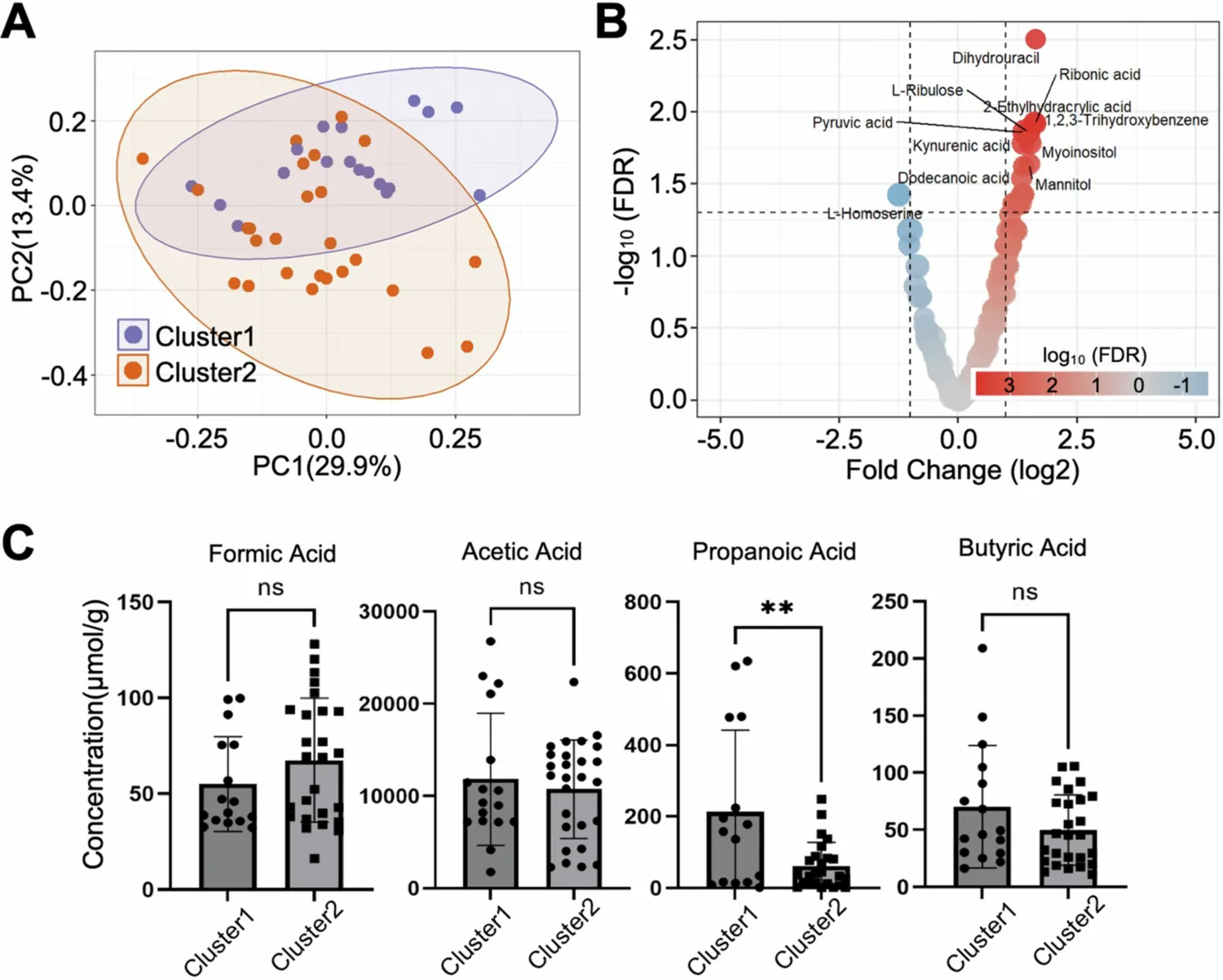
(A) PCA plot illustrating variance in the fecal metabolome between Cluster 1 and Cluster 2. (B) Volcano plot showing metabolites that differed significantly between Cluster 1 and Cluster 2. Dashed lines indicate thresholds of |log₂ fold change| > 1 and FDR < 0.05. (C) Fecal concentrations of formic, acetic, propanoic, and butyric acids in Cluster 1 and Cluster 2, with significance assessed using the Wilcoxon rank-sum test.

## Discussion

This study demonstrated that dogs with CE and those with acute diarrhea exhibit distinct fecal microbiome profiles compared with healthy controls, characterized by reduced microbial diversity and altered community composition. Unsupervised PAM clustering of CE cases based on fecal microbiota profiles identified two biologically meaningful subgroups that differed in disease severity, treatment response, and fecal metabolomic profiles. Key taxa contributing to these differences included *Streptococcus*, *Enterococcus_H_360604*, *Clostridium_T*, *Phocaeicola_A_858004*, *Fusobacterium_A*, and *Blautia_A_141781*. Although no clear metabolomic separation was observed between the AD and CE groups at the group level, this likely reflects the substantial within-group heterogeneity of the CE cohort, as microbiome-based subgrouping subsequently revealed more pronounced metabolomic distinctions between the two CE clusters than between the AD and CE groups as a whole. These findings suggest that gut microbiota structure and associated metabolite profiles collectively contribute to variations in disease severity in canine CE.

Fecal microbiota analysis confirmed significant differences in community structure among healthy dogs, dogs with acute diarrhea, and those with CE, corroborating previous reports of distinct microbial profiles and reduced diversity in dogs with gastrointestinal disorders (AlShawaqfeh et al. 2017; Allenspach and Mochel 2022; Chen et al. 2025). Within the CE cohort, this pattern extended to the cluster level, with Cluster 2 showing lower alpha diversity than Cluster 1. At the phylum level, the core gut microbiota of healthy dogs was dominated by *Firmicutes*, *Fusobacteriota*, *Bacteroidota*, *Proteobacteria*, and *Actinobacteriota*, consistent with previous pyrosequencing-based reports (Hand et al. 2013). *Firmicutes* remained the most abundant phylum across all groups and encompasses taxa with well-established roles in SCFA production, including *Faecalibacterium* and *Blautia*, both of which have been reported to decline in dogs with CE (Pilla and Suchodolski 2020). *Bacteroidota* contributes primarily to polysaccharide degradation and acetate production (Mukhopadhya and Louis 2025), and its relative abundance has been reported to decrease during active intestinal inflammation (Shin et al. 2023; Ishikawa et al. 2024; Li et al. 2025). *Actinobacteriota*, which predominantly colonizes the small intestine, typically increases under conditions of elevated luminal oxygen, such as during acute diarrhea (Chung The and Le 2022). Notably, despite prolonged dysbiosis, the CE group exhibited a lower relative abundance of *Actinobacteriota* compared with the AD group, which may reflect an adaptive shift in the intestinal microenvironment over the chronic disease course. The CE group also showed the highest proportion of unassigned taxa, potentially indicating progressive erosion of the normal core microbiota.

In dogs with CE, fluctuations in specific bacterial taxa have been frequently documented (AlShawaqfeh et al. 2017; Jablonski Wennogle et al. 2017; Galler et al. 2022; Kaga et al. 2024). AlShawaqfeh et al. proposed the Dysbiosis Index (DI), which incorporates quantification of seven key taxa (*Faecalibacterium*, *Turicibacter*, *Streptococcus*, *Escherichia coli*, *Blautia*, *Fusobacterium*, and *Clostridium hiranonis*) to characterize gut microbial changes in CE dogs. Although our study employed 16S rRNA gene sequencing rather than the quantitative PCR-based DI method, the shifts in relative abundance we observed were broadly consistent with those described by the DI framework. In a related approach, Chen et al. (2025) stratified CE dogs by dysbiosis index status (elevated vs non-elevated) and identified distinct microbial gene and metabolite signatures in dogs with an elevated DI. Their approach relies on a predefined DI threshold derived from seven bacterial taxa, whereas the present study employed unsupervised PAM clustering to identify subtypes in a data-driven manner without a priori assumptions. Although the two approaches differ methodologically, both support the concept that CE dogs can be stratified into biologically meaningful microbiome-based subgroups. The unsupervised clustering used in the present study may complement the DI framework by capturing broader patterns of community structure beyond the specific taxa included in the DI panel. *Phocaeicola*, a Gram-negative anaerobic bacterium that constitutes approximately 11% of the gut microbiota in healthy dogs (Kitahara et al. 2005; Hand et al. 2013), showed reduced relative abundance in both CE clusters, with a more pronounced reduction in Cluster 2. Although its functional role in the canine gut remains incompletely characterized, its decline may reflect broader disruption of the normal core microbiota structure.

A particularly prominent feature of Cluster 2 was the markedly elevated relative abundance of *Streptococcus*. Increased *Streptococcus* abundance has been reported in dogs with both acute diarrhea and CE (Suchodolski et al. 2012; Galler et al. 2022; Bai et al. 2023), and its absolute fecal abundance is one of the criteria incorporated into the DI for assessing microbial dysbiosis in CE (AlShawaqfeh et al. 2017). LEfSe analysis confirmed that *Streptococcus* was the strongest discriminative taxon for Cluster 2 in the present study. Similarly, *Clostridium_T* was significantly enriched in Cluster 2; members of the *Clostridium* genus, including *Clostridium difficile*, are recognized pathogens associated with bacterial diarrhea in dogs (Marks and Kather 2003). The co-enrichment of these potentially pathogenic or opportunistic taxa suggests a further deterioration of the gut microbial environment in Cluster 2 animals.

Conversely, taxa associated with SCFA production were markedly depleted in Cluster 2. Multiple *Blautia* taxa showed reduced relative abundance in both CE clusters, with the most pronounced reduction in Cluster 2. *Blautia* is closely associated with butyrate synthesis and colonic mucus maintenance (Holmberg et al. 2024), and its decline has been documented in dogs with acute diarrhea and CE (Galler et al. 2022; Bai et al. 2023). Similarly, *Ruminococcus_B* was significantly reduced in Cluster 2; members of the Ruminococcaceae family contribute to acetate and butyrate production through resistant starch fermentation (Onodera et al. 2025). *Fusobacteria* abundance, which typically declines during active IBD in dogs and recovers during remission (Onodera et al. 2025), was also lower in Cluster 2. *Faecalibacterium*, a key butyrate producer that engages in cross-feeding interactions with *Bifidobacterium* to enhance SCFA synthesis (Sokol et al. 2008; Suchodolski et al. 2012; Onodera et al. 2025), was similarly reduced. Consistent with these microbiological findings, targeted SCFA analysis revealed that propanoic acid concentrations were significantly lower in Cluster 2 compared with Cluster 1. However, acetic and butyric acid concentrations did not differ significantly between clusters, despite the depletion of known butyrate and acetate producers such as *Fusobacterium*, *Blautia*, and *Faecalibacterium* in Cluster 2. This discrepancy may reflect functional redundancy within the gut microbial community, whereby other taxa partially compensate for the loss of specific SCFA producers. Additionally, SCFA concentrations are influenced not only by producer abundance but also by substrate availability, cross-feeding interactions, and intestinal transit time, which were not controlled in this study. The selective reduction in propanoic acid nonetheless suggests that propionate-producing pathways may be more vulnerable to the specific pattern of dysbiosis observed in Cluster 2. SCFAs, particularly propionate and butyrate, are essential for maintaining intestinal epithelial barrier integrity and modulating local immune responses (Minamoto et al. 2019), and even selective reductions may have functional consequences for intestinal homeostasis.

Taxa involved in bile acid and organic acid metabolism also showed notable alterations. *Alloprevotella* and *Anaerobiospirillum_A* were reduced in both CE clusters relative to the HC group, with further reductions in Cluster 2. *Alloprevotella* has been reported to decline during the disease phase of CE in dogs, and this reduction may be associated with impaired succinate metabolism, as succinate has been proposed as a potential biomarker of intestinal disease (Kaga et al. 2024). Certain *Anaerobiospirillum* species are known succinate producers (Nghiem et al. 1997), and their depletion may further perturb organic acid homeostasis.

Beyond SCFAs, untargeted metabolomics revealed elevated kynurenic acid levels in Cluster 2 relative to Cluster 1, suggesting enhanced kynurenine pathway activity. Tryptophan catabolism in dogs occurs predominantly via the kynurenine pathway, which accounts for at least 90% of tryptophan degradation (Yu et al. 2024). The remaining fraction is metabolized through the indole and serotonin pathways, both of which are closely linked to gut microbiota activity (Chajadine et al. 2024). Increased expression of indoleamine 2,3-dioxygenase 1 (IDO-1) has been documented in IBD and is associated with reduced circulating tryptophan concentrations (Kathrani et al. 2019; Churchhouse et al. 2024). Enhanced kynurenine pathway flux in Cluster 2 may therefore reduce tryptophan availability for the indole pathway, thereby diminishing the production of indole derivatives with anti-inflammatory properties and further compromising intestinal homeostasis. Although tryptophan and its direct metabolites were not measured in the present study, the elevated kynurenic acid levels observed in Cluster 2 provide indirect evidence of dysregulated tryptophan catabolism in the more severely dysbiotic cluster. Given that intestinal inflammation and dysbiosis have been shown to upregulate IDO-1 expression and activate the kynurenine pathway in murine colitis models (Zhao et al. 2023), the elevated kynurenic acid in Cluster 2 may reflect the greater overall severity of dysbiosis and intestinal inflammation in this subgroup rather than the effect of any single taxon.

This study has several limitations that should be considered when interpreting the findings. First, the relatively small sample sizes, particularly in the AD group (*n* = 8) and the treatment analysis subsets (Cluster 1: *n* = 12; Cluster 2: *n* = 18), limit the statistical power and generalizability of the findings; this may also account for the non-significant result in the ordinal logistic regression analysis of cluster–severity association. Nonetheless, the significant differences in remission rates and CCECAI changes between clusters support the biological relevance of the identified subtypes. Second, the cross-sectional and observational study design precludes causal inference between microbiome composition and disease outcomes, and treatment protocols were not standardized across cases; prospective studies with controlled interventions will be necessary to determine whether microbiome-based stratification can guide therapeutic decision-making. In addition, 41.1% of CE dogs had received steroids/immunosuppressants or antibiotics from their primary care veterinarians before referral and baseline fecal sampling, and detailed records of the timing of last administration were not consistently available. Although prior medication may have contributed to the overall dysbiotic profile of the CE cohort, the proportion of pre-medicated dogs did not differ between clusters (Cluster 1: 45.5% vs Cluster 2: 38.2%; *p* = 0.781), suggesting that the observed cluster-level differences are not attributable to differential medication exposure. Future prospective studies should ideally enroll treatment-naive patients or enforce standardized washout periods before microbiome sampling. Third, the use of relative rather than absolute abundances in microbiome profiling may introduce compositional bias (Gloor et al. 2017; Sung et al. 2023), and potential confounders such as breed, diet, and prior antibiotic or probiotic use were not systematically controlled. The HC group included puppies aged ≤ 3 months (*n* = 29), which may have influenced microbiota composition, as young dogs have been reported to harbor compositionally distinct microbiota with lower Faecalibacterium and Blautia and higher Streptococcus (Blake et al. 2020; Asaduzzaman et al. 2026). However, a sensitivity analysis excluding all puppies (retaining *n* = 97 adult dogs) confirmed that all key findings, including alpha and beta diversity differences and cluster-defining taxa, remained robust (Supplementary Figure S2).

Moreover, the primary conclusions of this study are based on within-CE-cohort comparisons, which are independent of the HC group composition. The consistency of our findings with established dysbiosis patterns in canine CE and the concordance between microbiome and metabolomic data provide converging evidence that supports the robustness of the observed cluster differences.

In conclusion, this study demonstrated that fecal microbiome profiles differ markedly among healthy dogs, those with acute diarrhea, and those with CE. Within the CE cohort, PAM clustering identified two distinct microbiome-based subtypes that differed in alpha and beta diversity, community composition, fecal metabolomic profiles, and clinical outcomes. Cluster 2, characterized by enrichment of potentially pathogenic taxa and depletion of SCFA producers, was associated with greater disease severity and a significantly lower remission rate following treatment among dogs with active disease at baseline (38.9% vs 90.0%; *p* = 0.0159). However, given the observational nature of this study, whether microbiome-based cluster assignment has true predictive value for treatment outcomes requires confirmation in prospective studies. These findings highlight the potential clinical value of microbiome-based patient stratification in canine CE and provide a foundation for future studies investigating targeted therapeutic strategies for distinct CE biotypes.

## Supplementary Material

Supplementary Material Supplementary figure caption

FigureS1.jpg

FigureS2.jpg FigureS2.jpg

Supplementary Table S4 1st.docx Supplementary Table S4 1st.docx

Supplementary Table S1 1st.docx Supplementary Table S1 1st.docx

Supplementary Table S2 1st.docx Supplementary Table S2 1st.docx

Supplementary Table S3 1st.xlsx Supplementary Table S3 1st.xlsx

## Data Availability

The datasets displayed in the present investigation can be found in online repositories. The repository/repositories’ names and the accession number(s) can be attained below: https://www.ncbi.nlm.nih.gov/genbank/, PRJNA1438898.

## References

[cit0001] Allenspach K , Mochel JP . 2022. Current diagnostics for chronic enteropathies in dogs. Vet Clin Pathol. 50:18–28. 10.1111/vcp.13068 34699081

[cit0002] Allenspach K , Wieland B , Gröne A , Gaschen F . 2007. Chronic enteropathies in dogs: evaluation of risk factors for negative outcome. J Vet Intern Med. 21:700–708. 10.1111/j.1939-1676.2007.tb03011.x 17708389

[cit0003] AlShawaqfeh MK et al. 2017. A dysbiosis index to assess microbial changes in fecal samples of dogs with chronic inflammatory enteropathy. FEMS Microbiol Ecol. 93:fix136. 10.1093/femsec/fix136 29040443

[cit0004] Andoh A , Nishida A . 2022. Alteration of the gut microbiome in inflammatory bowel disease. Digestion. 104:16–23. 10.1159/000525925 35901721

[cit0005] Asaduzzaman M et al. 2026. Longitudinal long-read microbiome profiling in a canine model reveals how age, diet, and birth mode shape gut community dynamics. mSphere. 11:e0127925. 10.1128/msystems.01279-25 PMC12911399 41575196

[cit0006] Bai H , Liu T , Wang S , Shen L , Wang Z . 2023. Variations in gut microbiome and metabolites of dogs with acute diarrhea in poodles and labrador retrievers. Arch Microbiol. 205:97. 10.1007/s00203-023-03439-6 36823480

[cit0007] Blake AB et al. 2020. Developmental stages in microbiota, bile acids, and clostridial species in healthy puppies. J Vet Intern Med. 34:2345–2356. 10.1111/jvim.15928 PMC7694855 33047396

[cit0008] Bolyen E et al. 2019. Reproducible, interactive, scalable and extensible microbiome data science using QIIME 2. Nat Biotechnol. 37:852–857. 10.1038/s41587-019-0209-9 PMC7015180 31341288

[cit0009] Callahan BJ , McMurdie PJ , Rosen MJ , Han AW , Johnson AJA , Holmes SP . 2016. DADA2: high-resolution sample inference from illumina amplicon data. Nat Methods. 13:581–583. 10.1038/nmeth.3869 PMC4927377 27214047

[cit0010] Chajadine M et al. 2024. Harnessing intestinal tryptophan catabolism to relieve atherosclerosis in mice. Nat Commun. 15:6390. 10.1038/s41467-024-50807-x PMC11289133 39080345

[cit0011] Chen C-C et al. 2025. Microbial gene profiling and targeted metabolomics in fecal samples of dogs with chronic enteropathy with or without increased dysbiosis index. J Vet Intern Med. 39:e70199. 10.1111/jvim.70199 PMC12351495 40810386

[cit0012] Chung The H , Le S-NH . 2022. Dynamic of the human gut microbiome under infectious diarrhea. Curr Opin Microbiol. 66:79–85. 10.1016/j.mib.2022.01.006 PMC9758627 35121284

[cit0013] Churchhouse AMD et al. 2024. Loss of DOCK2 potentiates inflammatory bowel disease–associated colorectal cancer via immune dysfunction and IFNγ induction of IDO1 expression. Oncogene. 43:3094–3107. 10.1038/s41388-024-03135-9 PMC11473400 39242821

[cit0014] Dandrieux JRS . 2016. Inflammatory bowel disease versus chronic enteropathy in dogs: are they one and the same? J Small Anim Pract. 57:589–599. 10.1111/jsap.12588 27747868

[cit0015] Dandrieux JRS , Mansfield CS . 2019. Chronic enteropathy in canines: prevalence, impact and management strategies. Vet Med Res Rep. 10:203–214. 10.2147/VMRR.S162774 PMC6902862 31828025

[cit0016] Drost H-G . 2018. Philentropy: information theory and distance quantification with R. J Open Source Softw. 3:765. 10.21105/joss.00765

[cit0017] Franzosa EA et al. 2019. Gut microbiome structure and metabolic activity in inflammatory bowel disease. Nat Microbiol. 4:293–305. 10.1038/s41564-018-0306-4 PMC6342642 30531976

[cit0018] Fritsch DA et al. 2022. Microbiome function underpins the efficacy of a fiber-supplemented dietary intervention in dogs with chronic large bowel diarrhea. BMC Vet Res. 18:245. 10.1186/s12917-022-03315-3 PMC9233311 35751094

[cit0019] Furuhashi T , Sugitate K , Nakai T , Jikumaru Y , Ishihara G . 2018. Rapid profiling method for mammalian feces short chain fatty acids by GC-MS. Anal Biochem. 543:51–54. 10.1016/j.ab.2017.12.001 29221706

[cit0020] Gacesa R et al. 2022. Environmental factors shaping the gut microbiome in a Dutch population. Nature. 604:732–739. 10.1038/s41586-022-04567-7 35418674

[cit0021] Galler AI et al. 2022. Microbial dysbiosis and fecal metabolomic perturbations in yorkshire terriers with chronic enteropathy. Sci Rep. 12:12977. 10.1038/s41598-022-17244-6 PMC9334271 35902689

[cit0022] Gloor GB , Macklaim JM , Pawlowsky-Glahn V , Egozcue JJ . 2017. Microbiome datasets are compositional: and this is not optional. Front Microbiol. 8:2224. 10.3389/fmicb.2017.02224 PMC5695134 29187837

[cit0023] Guard BC et al. 2015. Characterization of microbial dysbiosis and metabolomic changes in dogs with acute diarrhea. PLoS One. 10:e0127259. 10.1371/journal.pone.0127259 PMC4441376 26000959

[cit0024] Hand D , Wallis C , Colyer A , Penn CW . 2013. Pyrosequencing the canine faecal microbiota: breadth and depth of biodiversity. PLoS One. 8:e53115. 10.1371/journal.pone.0053115 PMC3561364 23382835

[cit0025] Hennig C . 2024. fpc: flexible procedures for clustering. R package version 2.2-13. https://CRAN.R-project.org/package=fpc

[cit0026] Holmberg SM et al. 2024. The gut commensal blautia maintains colonic mucus function under low-fiber consumption through secretion of short-chain fatty acids. Nat Commun. 15:3502. 10.1038/s41467-024-47594-w PMC11045866 38664378

[cit0027] Ishikawa D et al. 2024. Anti-inflammatory effects of bacteroidota strains derived from outstanding donors of fecal microbiota transplantation for the treatment of ulcerative colitis. Inflamm Bowel Dis. 30:2136–2145. 10.1093/ibd/izae080 38733623

[cit0028] Jablonski Wennogle SA , Priestnall SL , Webb CB . 2017. Histopathologic characteristics of intestinal biopsy samples from dogs with chronic inflammatory enteropathy with and without hypoalbuminemia. J Vet Intern Med. 31:371–376. 10.1111/jvim.14669 PMC5354060 28256026

[cit0029] Jergens AE , Heilmann RM . 2022. Canine chronic enteropathy—current state-of-the-art and emerging concepts. Front Vet Sci. 9:923013. 10.3389/fvets.2022.923013 PMC9534534 36213409

[cit0030] Kaga C et al. 2024. Characterization of faecal microbiota and serum inflammatory markers in dogs diagnosed with chronic enteropathy or small-cell lymphoma: a pilot study. Sci Rep. 14:19387. 10.1038/s41598-024-69923-1 PMC11339456 39169196

[cit0031] Kathrani A et al. 2019. Indoleamine-pyrrole 2,3-dioxygenase-1 (IDO-1) mRNA is over-expressed in the duodenal mucosa and is negatively correlated with serum tryptophan concentrations in dogs with protein-losing enteropathy. PLoS One. 14:e0218218. 10.1371/journal.pone.0218218 PMC6557522 31181125

[cit0032] Kitahara M , Sakamoto M , Ike M , Sakata S , Benno Y . 2005. Bacteroides plebeius sp. Nov. and bacteroides coprocola sp. Nov., isolated from human faeces. Int J Syst Evol Microbiol. 55:2143–2147. 10.1099/ijs.0.63788-0 16166722

[cit0033] Komatsu Y et al. 2022. Dynamic associations of milk components with the infant gut microbiome and fecal metabolites in a mother–infant model by microbiome, NMR metabolomic, and time-series clustering analyses. Front Nutr. 8:813690. 10.3389/fnut.2021.813690 PMC8780135 35071301

[cit0034] Li J et al. 2024. Changes in fecal microbiota during estrous cycle in healthy thoroughbred mares. J Equine Vet Sci. 135:105034. 10.1016/j.jevs.2024.105034 38428754

[cit0035] Li R , Sun Y , Kelsang D , Feng M , Cai H , Zhang J . 2025. Butyrate ameliorates DSS-induced ulcerative colitis in mice by facilitating autophagy in intestinal epithelial cells and modulating the gut microbiota through blocking the PI3K-AKT-mTOR pathway. PLoS One. 20:e0337214. 10.1371/journal.pone.0337214 PMC12697976 41379810

[cit0036] Liu C et al. 2025. A workflow for statistical analysis and visualization of microbiome omics data using the R microeco package. Nat Protoc. 21:1300–1324. 10.1038/s41596-025-01239-4 40770112

[cit0037] Maechler M , Rousseeuw P , Struyf A , Hubert M , Hornik K . 2019. cluster: cluster analysis basics and extensions. R package.

[cit0038] Makowski D . 2018. The psycho package: an efficient and publishing-oriented workflow for psychological science. J Open Source Softw. 3(22):470. 10.21105/joss.00470

[cit0039] Marks SL , Kather EJ . 2003. Antimicrobial susceptibilities of canine clostridium difficile and clostridium perfringens isolates to commonly utilized antimicrobial drugs. Vet Microbiol. 94:39–45. 10.1016/S0378-1135(03)00061-0 12742714

[cit0040] McDonald D et al. 2024. Greengenes2 unifies microbial data in a single reference tree. Nat Biotechnol. 42:715–718. 10.1038/s41587-023-01845-1 PMC10818020 37500913

[cit0041] Minamoto Y et al. 2019. Fecal short-chain fatty acid concentrations and dysbiosis in dogs with chronic enteropathy. J Vet Intern Med. 33:1608–1618. 10.1111/jvim.15520 PMC6639498 31099928

[cit0042] Mukhopadhya I , Louis P . 2025. Gut microbiota-derived short-chain fatty acids and their role in human health and disease. Nat Rev Microbiol. 23:635–651. 10.1038/s41579-025-01183-w 40360779

[cit0043] Nghiem NP , Davison BH , Suttle BE , Richardson GR . 1997. Production of succinic acid by anaerobiospirillum succiniciproducens. Appl Biochem Biotechnol. 63:565–576. 10.1007/BF02920454 18576111

[cit0044] Onodera H , Sato Y , Komatsu Y , Yamashita M , Watanabe Y , Kokubo T . 2025. HMOs induce butyrate production of faecalibacterium prausnitzii via cross-feeding by bifidobacterium bifidum with different mechanisms for HMO types. Microorganisms. 13:1705. 10.3390/microorganisms13071705 PMC12298403 40732214

[cit0045] Pilla R , Suchodolski JS . 2020. The role of the canine gut microbiome and metabolome in health and gastrointestinal disease. Front Vet Sci. 6:498. 10.3389/fvets.2019.00498 PMC6971114 31993446

[cit0046] Rodrigues SD et al. 2025. Association of diet with treatment response in dogs with chronic enteropathy: a retrospective multicenter study. J Vet Intern Med. 39:e70071. 10.1111/jvim.70071 PMC12053874 40326642

[cit0047] Shin Y et al. 2023. Roles of short-chain fatty acids in inflammatory bowel disease. Nutrients. 15:4466. 10.3390/nu15204466 PMC10609902 37892541

[cit0048] Shoubridge AP et al. 2022. The gut microbiome and mental health: advances in research and emerging priorities. Mol Psychiatry. 27:1908–1919. 10.1038/s41380-022-01479-w 35236957

[cit0049] Sokol H et al. 2008. Faecalibacterium prausnitzii is an anti-inflammatory commensal bacterium identified by gut microbiota analysis of crohn disease patients. Proc Natl Acad Sci. 105:16731–16736. 10.1073/pnas.0804812105 PMC2575488 18936492

[cit0050] Suchodolski JS et al. 2012. The fecal microbiome in dogs with acute diarrhea and idiopathic inflammatory bowel disease. PLoS One. 7:e51907. 10.1371/journal.pone.0051907 PMC3530590 23300577

[cit0051] Sung C-H et al. 2023. Correlation between targeted qPCR assays and untargeted DNA shotgun metagenomic sequencing for assessing the fecal microbiota in dogs. Animals. 13:2597. 10.3390/ani13162597 PMC10451198 37627387

[cit0052] Yu J et al. 2023. Serum proteome of dogs with chronic enteropathy. J Vet Intern Med. 37:925–935. 10.1111/jvim.16682 PMC10229361 37186013

[cit0053] Yu F et al. 2024. Association between metabolites in tryptophan-kynurenine pathway and inflammatory bowel disease: a two-sample mendelian randomization. Sci Rep. 14:201. 10.1038/s41598-023-50990-9 PMC10761717 38167867

[cit0054] Zhao L-P et al. 2023. DSS-induced colitis activates the kynurenine pathway in serum and brain by affecting IDO-1 and gut microbiota. Front Immunol. 13:1089200. 10.3389/fimmu.2022.1089200 PMC9908955 36776388

[cit0055] Zheng J et al. 2024. Noninvasive, microbiome-based diagnosis of inflammatory bowel disease. Nat Med. 30:3555–3567. 10.1038/s41591-024-03280-4 PMC11645270 39367251

