# Supplementary material for: Fecal microbiome-based clustering identifies distinct disease subtypes with differing metabolomic profiles and treatment outcomes in canine chronic enteropathy
Source: Vet Q. 2026 Oct 29;46(1):2744638. doi: 10.1080/01652176.2026.2744638 (PMC13726330; doi:10.1080/01652176.2026.2744638)
Supplement: Supplementary file 1 [file TVEQ_A_2744638_SM3278.docx]

**Supplementary Figure S1.** Calinski–Harabasz index values for k = 2 to 20 clusters, used to determine the optimal number of PAM clusters from fecal microbiota profiles of dogs with chronic enteropathy.

**Supplementary Figure S2.** Sensitivity analysis excluding puppies aged ≤ 3 months from the healthy control group (HC, n = 97 adult dogs). (A) Comparison of fecal microbiota α-diversity (Chao1 index, upper; Shannon index, lower) among HC, AD, and CE groups. (B) PCoA of fecal microbiota β-diversity based on Bray–Curtis dissimilarity (PERMANOVA F = 20.907, R² = 0.208, p = 0.001).
