# Supplementary material for: Fecal microbiome-based clustering identifies distinct disease subtypes with differing metabolomic profiles and treatment outcomes in canine chronic enteropathy
Source: Vet Q. 2026 Oct 29;46(1):2744638. doi: 10.1080/01652176.2026.2744638 (PMC13726330; doi:10.1080/01652176.2026.2744638)
Supplement: Supplementary file 2 [file TVEQ_A_2744638_SM3657.docx]

**Supplementary Table S2. Individual information of dogs in the acute diarrhea (AD) group.**

| **Sample ID** | **Breed** | **Age** | **Probiotic/Supplement use** | **Concurrent medication** |
| --- | --- | --- | --- | --- |
| OAC-001 | Yorkshire Terrier | 1 yr 8 mo | No | None |
| OAC-002 | Labrador Retriever (puppy) | 2 mo | Yes (Miya BM) | None |
| OAC-003 | Pekingese | 1 yr 8 mo | No | None |
| OAC-004 | Mixed breed | 11 yr 6 mo | No | None |
| OAC-006 | Cavalier King Charles Spaniel | 8 yr | No | Apoquel |
| OAC-007 | Shih Tzu | 16 yr 2 mo | No | None |
| OAC-009 | Mix (Maltese × Chihuahua) | 8 yr 1 mo | No | None |
| OAC-011 | Mixed breed | 3 yr 4 mo | No | None |

Eight dogs were included in the analysis. Samples OAC-005 and OAC-008, collected during recovery from the same individuals as OAC-003 and OAC-004, respectively, were excluded from all analyses.
