# Supplementary material for: Fecal microbiome-based clustering identifies distinct disease subtypes with differing metabolomic profiles and treatment outcomes in canine chronic enteropathy
Source: Vet Q. 2026 Oct 29;46(1):2744638. doi: 10.1080/01652176.2026.2744638 (PMC13726330; doi:10.1080/01652176.2026.2744638)
Supplement: Supplementary file 3 [file TVEQ_A_2744638_SM3658.docx]

**Supplementary Table S1. Breed composition of dogs in the healthy control (HC) group.**

| **Breed** | **Adult dogs (n)** | **Puppies (n)** | **Total (n)** |
| --- | --- | --- | --- |
| Chihuahua | 8 | 4 | 12 |
| Toy Poodle | 12 | 4 | 16 |
| Dachshund | 8 | 4 | 12 |
| Maltese | 11 | 4 | 15 |
| Yorkshire Terrier | 7 | 5 | 12 |
| Shiba Inu | 9 | 1 | 10 |
| Schnauzer | 4 | 1 | 5 |
| West Highland White Terrier | 15 | 0 | 15 |
| Miniature Dachshund | 3 | 0 | 3 |
| Cavalier King Charles Spaniel | 2 | 0 | 2 |
| Pekingese | 16 | 2 | 18 |
| Bichon Frisé | 2 | 4 | 6 |
| **Total** | **97** | **29** | **126** |

A total of 126 dogs were enrolled from six commercial breeding facilities. Puppies were defined as dogs aged ≤ 3 months at the time of sample collection.
