# Supplementary material for: Fecal microbiome-based clustering identifies distinct disease subtypes with differing metabolomic profiles and treatment outcomes in canine chronic enteropathy
Source: Vet Q. 2026 Oct 29;46(1):2744638. doi: 10.1080/01652176.2026.2744638 (PMC13726330; doi:10.1080/01652176.2026.2744638)
Supplement: Supplementary file 4 [file TVEQ_A_2744638_SM3659.docx]

**Supplementary Table S4. Clinical and demographic characteristics of CE dogs in the treatment analysis subset (baseline CCECAI > 3), stratified by microbiome cluster.**

|  |  | **Cluster 1 (n = 12)** | **Cluster 2 (n = 18)** | **p-value** |
| --- | --- | --- | --- | --- |
| Body weight (kg) | Median (IQR) | 4.30 (3.31–6.86) | 5.75 (3.92–8.14) | 0.320 |
| Serum albumin (g/dL) | Median (IQR) | 2.35 (1.85–2.60) | 2.00 (1.45–2.10) | 0.182 |
| CRP (mg/dL) | Median (IQR) | 1.30 (0.39–5.38) | 0.73 (0.26–1.41) | 0.253 |
| Hypoalbuminemia | Albumin < 2.0 g/dL | 5/12 (41.7%) | 8/18 (44.4%) | 1.000 |
| Pre-medicationᵃ | Yes | 5/12 (41.7%) | 8/18 (44.4%) | 1.000 |
| Baseline CCECAI | Median (IQR) | 7.0 (4.0–8.2) | 7.5 (5.0–13.0) | 0.366 |
| Mediation^b^ | S/I  AB  PB  other | 9/12 (75.0%)  7/12 (58.3%)  0/12 (0.0%)  7/12 (58.3%) | 17/18 (94.4%)  12/18 (66.7%)  1/18 (5.6%)  11/18 (61.1%) | 0.274  0.712  1.000  1.000 |

Continuous variables compared using Mann–Whitney U test; categorical variables compared using Fisher's exact test.

ᵃ Pre-medication: dogs receiving steroids/immunosuppressants (S/I) or antibiotics (AB) before baseline fecal sampling.

^b^ Medication administered between initial and follow-up CCECAI assessments; dogs may fall into multiple categories. NR cases excluded from denominators.

Data shown as median (IQR) or n/total (%). BW, body weight; ALB, serum albumin; CRP, C-reactive protein; CCECAI, Canine Chronic Enteropathy Clinical Activity Index.
